# Interplay between epigenetics and metabolism controls cancer stem cell plasticity

**DOI:** 10.3389/freae.2024.1424163

**Published:** 2024-09-11

**Authors:** Jee-Eun Choi, Inwha Baek

**Affiliations:** ^1^ Department of Pathology, School of Clinical Medicine, The University of Hong Kong, Pokfulam, Hong Kong SAR, China; ^2^ College of Pharmacy, Kyung Hee University, Seoul, Republic of Korea; ^3^ Department of Regulatory Science, Graduate School, Kyung Hee University, Seoul, Republic of Korea; ^4^ Institute of Regulatory Innovation through Science (IRIS), Kyung Hee University, Seoul, Republic of Korea

**Keywords:** epigenetics, cancer stem cell (CSC), cancer metabolism, oncogenic reprogramming, cellular plasticity, intratumor heterogeneity

## Abstract

Tumors consist of cancer cells with different genetic, epigenetic, and phenotypic properties. Cancer stem cells are an important subpopulation of heterogeneous cancer cells and are capable of initiating and propagating tumors. The term cancer stem cells has become broader in efforts to understand their phenotypic plasticity to switch fates between self-renewal and differentiation. Cancer stem cell plasticity is significantly associated with the initiation of metastasis, resistance to therapy, and tumor recurrence. With our broadened knowledge of epigenetic regulation and metabolic reprogramming as key elements enabling such capabilities, an expansive body of literature has demonstrated the functional importance of each element in contributing to cancer stem cell characteristics. Recently, the direct interplay between epigenetic regulation and metabolic reprogramming has begun to be appreciated in the context of cancer stem cells with growing interest. In this review, we discuss the mechanisms by which cancer stem cells orchestrate the reciprocal regulation of cellular metabolism and epigenetic alterations. In the discussion, compelling, unanswered questions on this topic have been elaborated for the interest of the research community and how recent technological developments help tackle such research ideas. A comprehensive understanding of cancer stem cell attributes that are largely governed by epigenetic and metabolic reprogramming would enable the advancement of precise therapeutic options and the prediction of better responses to drugs, holding great promise in cancer treatment and cure.

## 1 Introduction

Tumor heterogeneity arises from multiple dimensions, including genetic and non-genetic levels with spatial and temporal differences. Our increasing understanding of tumor heterogeneity sheds light on cancer stem cells (CSCs), a subpopulation of cancer cells that demonstrate a higher capacity for tumor initiation, progression, metastasis, epithelial-mesenchymal plasticity, and drug resistance (Kreso and Dick, 2014; Nassar and Blanpain, 2016; Oskarsson et al., 2014). In addition to genetic heterogeneity within the tumor mass (Dagogo-Jack and Shaw, 2018), different non-genetic events can play functional roles in such capabilities in CSCs (Easwaran et al., 2014; Kim and DeBerardinis, 2019). Metabolic and epigenetic states are the two key non-genetic elements that contribute to CSC characteristics.

The interplay between metabolism and epigenetics in a pool of cancer cells has been extensively studied over the past decade at the population-averaged level (Izzo et al., 2021). In this review, we focus on the direct interplay between metabolic and epigenetic regulations in CSCs, which is a growing area of interest. Potential future research questions that are yet to be answered will be discussed to highlight the significance of this research area, which directly affects the therapeutic response and recurrence rate of patients with cancer.

## 2 CSCs: a minor but functionally critical subpopulation of cancer cells

Tumors are heterogeneous, as they continuously evolve at multiple layers, including genetic and epigenetic alterations and metabolic reprogramming (Meacham and Morrison, 2013). Cell-intrinsic and cell-extrinsic models to explain intratumor heterogeneity strongly suggest the presence of a tumor subpopulation with a unique ability to initiate and propagate tumors, metastasize with epithelial-mesenchymal plasticity, relapse post-therapy, and develop insensitivity to anti-cancer drugs, now known as CSCs (Kreso and Dick, 2014; Nassar and Blanpain, 2016; Oskarsson et al., 2014) (Figure 1). They are termed as such because of their functional resemblance to normal stem cells, which are characterized by self-renewal and differentiation potentials. Notably, CSCs were first identified in human acute myeloid leukemia (AML) by Bonnet and Dick based on their ability to propagate primary tumors and recreate phenotypic heterogeneity (Bonnet and Dick, 1997; Lapidot et al., 1994). Since pioneering studies, an increasing body of evidence has supported the existence of CSCs in various cancer types, such as breast cancer (Al-Hajj et al., 2003; Ginestier et al., 2007), colorectal cancer (O'Brien et al., 2007; Ricci-Vitiani et al., 2007), and glioblastoma (Singh et al., 2004).

**FIGURE 1:**
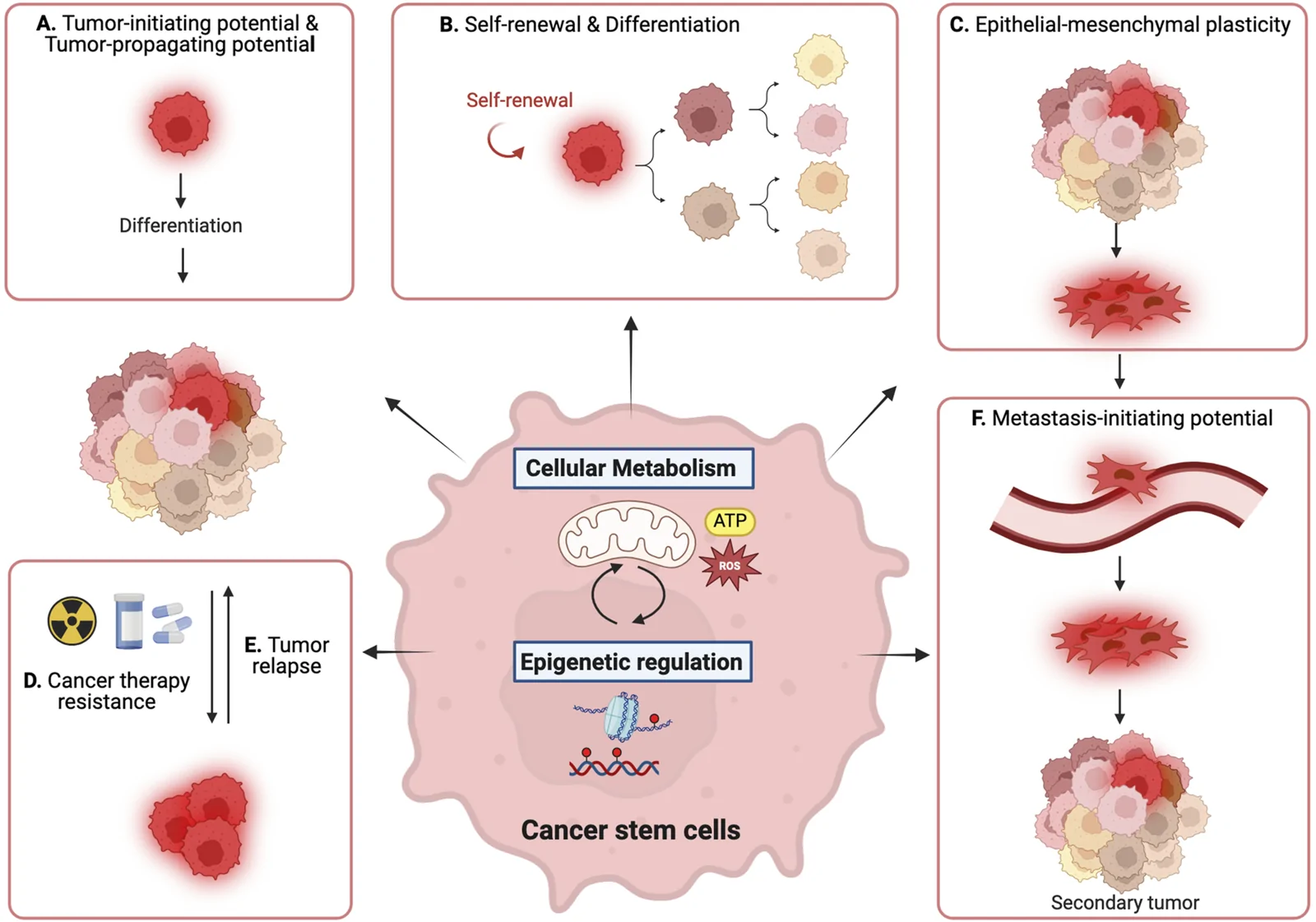
Reciprocal interaction between metabolic pathways and epigenetic regulation governs cancer stem cell (CSC) properties. **(A)** tumor-initiating and -propagating potential, **(B)** self-renewal and differentiation, **(C)** epithelial-mesenchymal plasticity, **(D)** cancer therapy resistance, **(E)** tumor relapse, and **(F)** metastasis-initiating potential.

Furthermore, CSCs are critical targets for anti-cancer therapeutics because of their distinctive roles in metastasis, therapy resistance, and tumor relapse. These abilities are mainly attributed to their phenotypic plasticity, including dedifferentiation and transdifferentiation (Pérez-González et al., 2023). In addition to cell-autonomous dysregulation of cell fate, CSCs can change their fate in response to environmental cues, similar to normal tissue stem cells (Batlle and Clevers, 2017). This dynamic cell fate transition involves the reprogramming of metabolic and epigenetic processes, which have been recognized as cancer hallmarks (Hanahan, 2022). This metabolic transition is thought to be partially achieved through epigenetic regulation. Conversely, metabolic alterations can affect epigenetic states. Metabolism and epigenetic regulation are not distinct but rather intricately connected as important reciprocal regulators of CSC plasticity, orchestrating CSC identity and characteristics. A comprehensive understanding of these two regulatory circuits in CSCs would enable the eradication of aggressive tumor cells and enhance tumor-free survival.

## 3 Metabolism controls epigenetics in CSCs

Metabolic rewiring is a well-known adaptation of cancer cells that provides survival and growth advantages in response to a tumor microenvironment with limited nutrients and oxygen and with stress (Faubert et al., 2020). Metabolic reprogramming during carcinogenesis and metastasis has been studied extensively over the last two decades (Pavlova and Thompson, 2016). Recently, metabolic heterogeneity has begun to be appreciated within the cancer cell population, along with the discovery of different cell surface markers and the development of metabolite measurement technologies for cellular resolution (Demicco et al., 2024). Remarkably, the metabolic phenotypes of CSCs differ from those of non-CSCs, raising exciting research questions regarding the functional roles of distinct metabolic pathways in CSCs.

Metabolic intermediates can serve as direct substrates or cofactors for the modifications of epigenetic marks (Wellen et al., 2009; Shyh-Chang et al., 2013; Carey et al., 2015). As epigenetic marks can be dynamically regulated during embryonic stem cell (ESC) differentiation, partly under the influence of metabolic changes, the metabolic state is a crucial determinant of cell lineage and identity (Ryall et al., 2015; Moussaieff et al., 2015; Sperber et al., 2015; Etchegaray and Mostoslavsky, 2016). During carcinogenesis, cell fate changes, partly through chromatin rearrangements and DNA/RNA modifications, favoring oncogenic reprogramming. As direct substrates or cofactors of enzymes responsible for epigenetic modifications, metabolites can regulate such phenotypes via epigenetic alterations, affecting the tumor-initiating, tumor-propagating, or metastatic potential. In this section, the key metabolites are discussed individually, focusing on their roles in writing and erasing epigenetic marks in CSCs.

### 3.1 Metabolites that generate epigenetic marks in CSC regulation

Acetyl-CoA and S-adenosyl methionine (SAM) are the most well-studied substrates for donating acetyl and methyl groups, respectively, to create new epigenetic modifications (Figure 2) (Boon et al., 2020). Acetyl-CoA is mainly produced during glucose metabolism and fatty acid oxidation (FAO). An increase in glycolysis and FAO results in abundant acetyl-CoA, which augments levels of global histone acetylation (Sivanand et al., 2018). Conversely, a decrease in the glycolysis-mediated generation of acetyl-CoA leads to a reduction in histone acetylation and thus affects the differentiation of ESCs, which emphasizes that the levels of intracellular metabolites feeding into epigenetic modifications can govern the cell differentiation state (Moussaieff et al., 2015). Hypoxia-induced upregulation of genes involved in glucose metabolism, such as glucose transporter 1 (GLUT1), pyruvate kinase isozyme M2, and pyruvate dehydrogenase A, enhances glucose influx and pyruvate production in breast cancer (Yang D. et al., 2020). This results in acetyl-CoA accumulation, an increase in histone H4 acetylation, and subsequent gene upregulation associated with cancer stemness, affecting the tumor-initiating capacity (Yang D. et al., 2020). An FAO-mediated increase in acetyl-CoA can epigenetically regulate epithelial-mesenchymal transition (EMT) gene expression, thus affecting EMT-driven metastasis in breast cancer (Loo et al., 2021). The balance between FAO and fatty acid storage regulates intracellular acetyl-CoA availability, a critical epigenetic module affecting epithelial-mesenchymal plasticity. These results suggest that the cell metabolic state can affect gene expression by providing surplus materials for writing epigenetic marks, thereby promoting the CSC potential of cellular plasticity, such as epithelial and mesenchymal states. Moreover, dietary palmitic acid can establish a stable epigenetic memory, particularly mediated by Set1A-dependent H3K4me3 deposition, critical for metastasis initiation and development of the pro-metastatic niche (Pascual et al., 2021). In addition to histone modifications, post-translational acetylation of non-histone proteins, such as signaling proteins and transcription factors (TFs) can also regulate the metastatic potential of breast cancer cells. For example, the exogenous supply of saturated fatty acid, palmitate, a rich source of acetyl-CoA, acetylates nuclear factor-kappaB p65 subunit, leading to the promotion of metastasis-initiating potential of breast cancer to lung and liver (Altea-Manzano et al., 2023).

**FIGURE 2:**
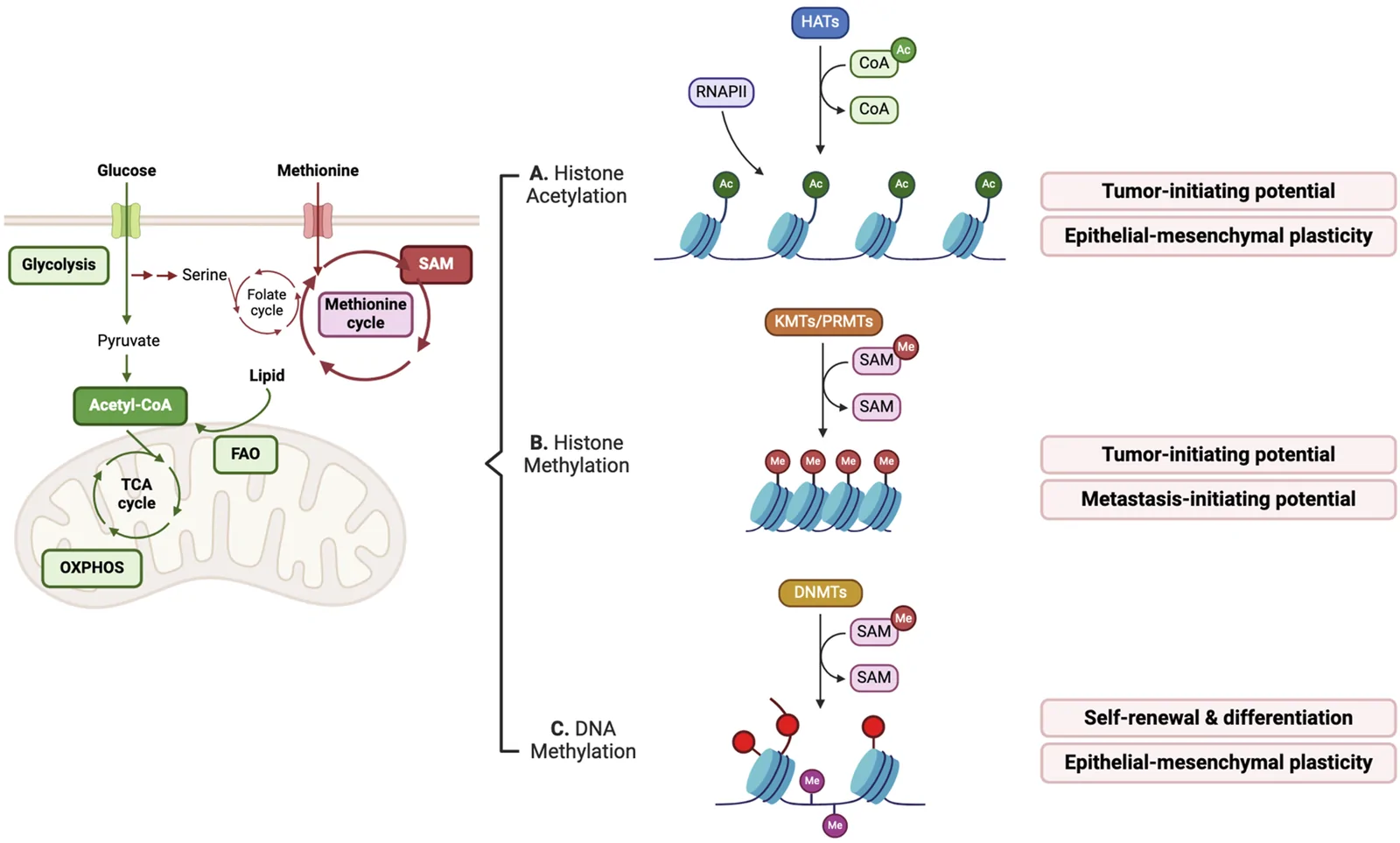
Metabolites serve as key substrates in producing epigenetic marks, thus affecting CSC properties. **(A)** Intracellular acetyl-CoA metabolism affects histone acetylation levels, in general promoting gene transcription associated with epithelial-mesenchymal transition (EMT) and enhancing tumor-initiating potential. **(B,C)** Intracellular S-Adenosyl methionine (SAM) abundance controls histone and DNA methylation states, which influences CSC features. RNAPII; RNA polymerase II.

As a methyl donor, SAM is involved in the creation of methylation marks on histone proteins, DNA and RNA. Aberrant regulations of glucose, glutamine, and serine/glycine metabolic pathways, and folate and methionine cycles have indirect and direct effects on the availability of intracellular SAM, thereby affecting the epigenetic modification of methyl group acceptors (Figure 2) (Mentch et al., 2015). Threonine and methionine, which are SAM-producing amino acids, can modulate histone methylation, thereby influencing ESC differentiation ability (Shyh-Chang et al., 2013; Shiraki et al., 2014). Histone methylation of lysine and arginine residues is mediated by lysine methyltransferases (KMTs) and protein arginine methyltransferases (PRMTs), respectively, which provide docking sites for epigenetic regulators (Stallcup, 2001). The number of covalent methyl groups in histones generates mono-, di-, and tri-methylation states and functions as regulatory signals that affect gene activation or repression, depending on the histone modifications at specific loci. In non-small cell lung cancer (NSCLC), tumor-initiating cells exhibit a unique metabolic dependence on methionine because of their demands for SAM. The key methionine cycle enzyme, methionine adenosyltransferase II alpha (MAT2A), plays a critical role in providing SAM for global increases in histone H3 methylation, such as H3K4me3, H3K9me3, H3K27me3, and H3K36me2/3, suggesting that the upregulation of histone methylation, regardless of active or repressive marks, can positively influence the tumor-initiating potential of NSCLC (Wang et al., 2019). In triple-negative breast cancer, restriction of SAM availability by MAT2A inhibition causes failure to maintain active H3K4me3 marks on the SOX9 gene locus, which is associated with CSC enrichment and tumor- and metastasis-initiating potential (Strekalova et al., 2019). In basal-like breast cancer, nicotinamide N-methyltransferase (NNMT), which negatively regulates SAM availability, can decrease H3K9me3 repressive marks and DNA methylation of the pro-metastatic genes, such as PR/SET domain-5, promoting basal-type cancer plasticity and metastasis-initiating potential by regulating H3K9me3 and DNA methylation (Couto et al., 2023).

DNA methylation occuring mainly at cytosine (5-methylcytosine [5-mC]) in cytosine-guanine (CpG) dinucleotides, is mediated by DNA methyltransferases (DNMTs), and regulates gene activation or repression by hypo or hypermethylation, respectively (Lyko, 2018). In glioblastoma stem cells (GSCs), increased NNMT expression depletes intracellular SAM levels, resulting in global DNA hypomethylation with a mesenchymal phenotype and the self-renewal of GSCs (Jung et al., 2017). The dependence of leukemia stem cells (LSCs) on oxidative phosphorylation (OXPHOS) in mitochondria links intracellular SAM availability to DNA methylation (Singh et al., 2020). An increase in mitochondrial copper under the inhibition of the mitochondria copper chaperone COX17 inhibits the enzymatic activity of S-adenosylhomocysteine hydrolase, thereby decreasing intracellular SAM levels (Singh et al., 2020). This specifically downregulates global DNA methylation but not histone methylation, which is essential for LSC viability and differentiation.

### 3.2 Metabolites that remove or regulate the removal of epigenetic marks in CSC regulation

Mitochondrial intermediates such as α -ketoglutarate ( α -KG), succinate, and fumarate function as direct cofactors or inhibitory molecules in removing epigenetic marks (Xiao et al., 2012; Killian et al., 2013; Letouzé et al., 2013). These metabolites center on regulating α -KG-mediated dioxygenase reactions, mainly removing methyl group(s) from histone, DNA, or RNA, thus affecting cancer initiation, progression, and plasticity (Figure 3) (Losman et al., 2020). Moreover, 2-hydroxyglutarate (2-HG), an oncometabolite produced by isocitrate dehydrogenase (IDH) gain-of-function mutations can also directly inhibit epigenetic enzymes such as histone and DNA demethylases utilizing α -KG as a cofactor (Figure 3) (Dang et al., 2009; Losman et al., 2013; Xu et al., 2011; Chowdhury et al., 2011).

**FIGURE 3:**
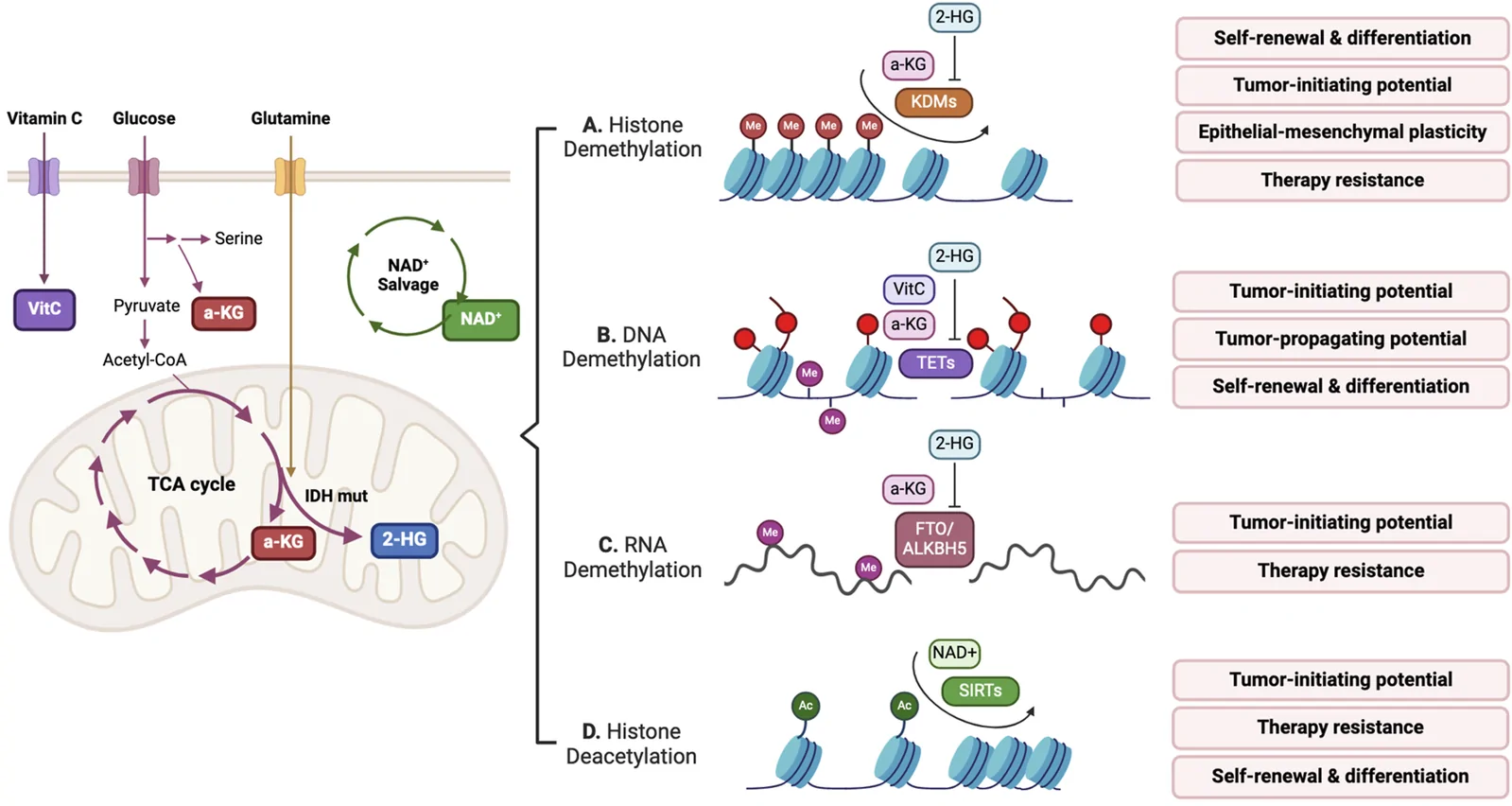
Metabolites take part in erasing epigenetic marks, which regulates CSC potential. **(A–C)** α -ketoglutarate ( α -KG)-dependent demethylation reactions of histone, DNA, and RNA are largely governed by intracellular levels of α -KG and 2-hydroxyglutarate (2-HG), an oncometabolite. Dynamic regulatory modules confer various traits to CSCs. **(D)** Sirtuins utilize NAD^+^ to remove acetyl group(s) from histone. This deacetylation affects cell differentiation, tumor initiation, and resistance to therapy.

Lysine demethylases (KDMs) catalyze the removal of methyl groups from histone lysine residues. Except for KDM1 (lysine-specific demethylase 1 [LSD1]), which utilizes flavin adenine dinucleotide (FAD) as a cofactor, the other KDMs (KDM2–7, JmjC domain-containing histone demethylases) are α -KG-dependent lysine demethylases targeting H3K4, H3K9, H3K27, and H3K36 (Kooistra and Helin, 2012; Klose et al., 2006). Local nutrient availability in the microenvironment can modulate histone demethylase activity by affecting α -KG levels. In melanoma, limited glutamine availability in tumor core regions led to decreased α -KG levels, resulting in histone H3K27 hypermethylation through insufficient ability to activate the H3K27-specific demethylase KDM6B (Pan et al., 2016). This H3K27 hypermethylation results in a dedifferentiation phenotype and therapeutic resistance to BRAF inhibitors, which are key characteristics of CSCs. In squamous cell carcinoma, activation of *de novo* serine synthesis pathway upon limited exogenous serine promotes α -KG-dependent H3K27me3 demethylation, thus facilitating cell differentiation and antagonizing tumor initiation (Baksh et al., 2020). In lung adenocarcinoma, reduction in glucose availability and hence in α -KG levels caused hypermethylation of H3K4me3, H3K9me3, and H3K27me3 (Saggese et al., 2024). An increase in H3K27me3 repressive marks was observed for genes associated with epithelial differentiation. In contrast, an increase in H3K4me3 activation marks was observed in the promoter regions enriched in canonical EMT TFs such as *SNAI1*, *SLUG*, and *ZEB1*. Therefore, understanding how different nutrient availability within different tumor microenvironments converges into intracellular α -KG levels, while α -KG levels can be differentially translated to modulate demethylase reactions of histone at different lysine residues or of DNA/RNA will be of considerable interest. The oncometabolite, 2-HG, can affect cell differentiation and stemness by inhibiting α -KG-dependent demethylation. Furthermore, the IDH gain-of-function mutation produces *R*-2-HG, which mainly inhibits the demethylation of H3K9me3 and H3K27me3 associated with cellular differentiation genes and thus blocks cell differentiation, promoting carcinogenesis in glioma and leukemia (Lu et al., 2012; Rohle et al., 2013; Wang F. et al., 2013)

Ten eleven translocation (TET) DNA demethylases belong to the α -KG-dependent dioxygenase enzyme family, catalyzing multiple oxidation reactions to remove the methyl group from 5-mC in DNA. Additionally, TET enzymatic activity is dysregulated in cancers with IDH mutations. In acute myeloid leukemia (AML) with IDH1 and IDH2 mutations, the global DNA hypermethylation phenotype is commonly induced by impaired TET2 function (Figueroa et al., 2010). Moreover, uniquely hypermethylated DNA regions were identified as GATA1/2 and EVI1 binding promoter regions, thereby inhibiting myeloid differentiation while maintaining stemness features. Branched-chain amino acids (BCAAs) undergo catabolism by transferring the α -amino group to α -KG via a BCAA transaminase (BCAT)-dependent reaction, which decreases intracellular α -KG. In AML stem cells, the BCAT1 enzyme and BCAA catabolic pathway are critical in regulating α -KG-dependent dioxygenase, including TET-mediated DNA demethylation (Raffel et al., 2017). Notably, BCAT1^high^ AML cells showed enrichment of leukemia stemness genes and a DNA hypermethylation signature with augmented tumor-initiating potential. In addition to α -KG, TET family enzymatic activity is regulated by ascorbate (Vitamin C). Among cancer types that frequently present loss-of-function mutations in TET1/2 enzymes, the intracellular levels of ascorbate significantly affect carcinogenesis and cancer cell differentiation. In leukemia, two independent research groups have reported that TET2 deficiency dysregulates stem cell self-renewal via aberrant DNA hypermethylation (Cimmino et al., 2017; Agathocleous et al., 2017). Moreover, ascorbate depletion, in conjunction with Flt3 alteration, can accelerate leukemia formation, mainly by regulating Tet2 enzymatic activity (Agathocleous et al., 2017). Ascorbate treatment of leukemia-induced TET-mediated DNA demethylation inhibits tumor-initiating potential and tumor progression (Cimmino et al., 2017).

RNA methylation of N^6^-methyladenosine (m^6^A) is a recently recognized epitranscriptomic mark of mRNA that affects the transcription, translation, alternative splicing, and stability of mRNA (Wang and Tang, 2023). Demethylation of m^6^A can be mediated by α -KG-dependent dioxygenase alk/B homolog 5 (ALKBH5) or fat mass and obesity-associated protein (FTO) as an α -KG-dependent enzymatic reaction. In breast cancer, hypoxia-induced ALKBH5 expression positively affects NANOG mRNA expression by its demethylation in the 3′-untranslated region (Zhang et al., 2016). Increased NANOG levels enhanced the breast CSC phenotype and tumor-initiating potential. In gliomas and leukemias harboring IDH1/2 mutations, *R*-2-HG can inhibit FTO activity, thereby increasing m^6^A RNA (Su et al., 2018). This compromises the stability of MYC/CEBPA transcripts by downregulating the FTO-mediated demethylase reaction, which may confer anti-tumor activity in IDH1/2-mutant cancers. In clear cell renal carcinoma, methionine derived from tumor-associated pericytes positively regulates the CSC phenotype through mRNA methylation (Zhang et al., 2024). An elevated SAM cycle specifically stabilizes ATPase-family-AAA-domain-containing 2 (ATAD2) mRNA via m^6^A modification, thereby promoting the formation of a super-enhancer complex with SOX9 and subsequent transcription of genes associated with CSC features.

An oxidized form of nicotinamide adenine dinucleotide, NAD^+^, functions as an essential cofactor in histone deacetylation and deacylation mediated by Sirtuins (SIRTs). Notably, SIRTs are a part of the histone deacetylase family and uniquely utilize NAD^+^, translating cellular metabolic and redox conditions into histone modifications, thereby affecting gene expression (Choi and Mostoslavsky, 2014). Furthermore, NAD^+^ serves as a substrate in reactions catalyzed by ADP-ribosyltransferases, such as poly (ADP-ribose) polymerase (PARP). In colorectal cancer, the NAD salvage pathway enzyme nicotinamide phosphoribosyl transferase (NAMPT) increases stemness and EMT-related gene expression, resulting in the enrichment of tumor-initiating cells through SIRT1 and PARP (Lucena-Cacace et al., 2018). In glioblastoma, NAMPT can regulate the NAD^+^-dependent transcriptional program targeted by E2F2-ID (inhibitor of differentiation) axis (Gujar et al., 2016). Intracellular NAD^+^ levels sustained by the NAMPT-mediated salvage pathway are key to GSC self-renewal and therapy resistance to therapy.

## 4 Epigenetics controls metabolism in CSCs

Epigenetic reprogramming has emerged as a cancer hallmark due to its role in initiating and sustaining malignant tumors (Hanahan, 2022). Exploiting epigenetic reprogramming is beneficial for CSCs, as it enables them to adapt to both extrinsic and intrinsic changes (Wainwright and Scaffidi, 2017; Easwaran et al., 2014). For example, transcriptional reprogramming governed by epigenetic regulation confers plasticity to phenotypic switching. Metabolic adaptation is a critical alteration that enables better survival and growth, and is partly mediated by epigenetic regulation (Miranda-Goncalves et al., 2018).

Epigenetic regulation occurs at various levels ranging from DNA methylation to histone modification and chromatin rearrangement. Epigenetic regulators can be grouped into *cis*-regulatory elements and *trans*-regulatory elements (Maston et al., 2006). *Cis*-regulatory elements are specialized DNA regions capable of regulating the expression of target genes, and include promoters, enhancers, silencers, and insulators (Preissl et al., 2023; Rivera and Ren, 2013). They typically serve as binding platforms for *trans*-regulatory elements. *Trans*-regulatory elements include TFs along with a broader range of DNA-binding proteins that contribute to gene expression regulation. Notably, TFs function by recruiting coactivators and enzymes that modulate the chromatin structure, thereby regulating the accessibility of specific DNA regions to the transcription machinery (Ferrie et al., 2022). These enzymes include histone modifiers, chromatin remodellers, and DNMTs. In this section, we focus on how the aforementioned epigenetic regulators orchestrate tumor metabolism to form and maintain CSC potential.

### 4.1 Transcription factors

Stem cell pluripotency and EMT TFs are often upregulated in CSCs (Yang L. et al., 2020; Chaudhary et al., 2023). They help CSCs maintain stem cell-like characteristics, partly by changing their metabolic state, preferentially shifting from OXPHOS to glycolysis and FAO (Figure 4) (Lisowski et al., 2018). In basal-like breast cancer, Snail (encoded by *SNAI1*), a critical EMT TF, forms a complex with a G9a histone methyltransferase and DNMT. The Snail-G9a-DNMT complex directly represses the expression of fructose-1,6-biphosphatase, a rate-limiting enzyme in gluconeogenesis. This results in increased glycolysis and reduced reactive oxygen species (ROS) production, conferring stem-like features to breast cancer cells (Dong et al., 2013). Tumor-initiating stem-like cells in hepatocellular carcinoma (HCC) promote self-renewal and support drug resistance through NANOG-mediated expression of FAO gene and concomitant repression of OXPHOS genes (Chen et al., 2016). *c-myc* is an essential TF in the thrombopoietin (TPO)-responsive colorectal CD110^+^ CSCs that promote liver metastasis. Notably, TPO-induced *c-myc* recruits the histone acetyltransferase HBO1, which increases the H3K14 acetylation of metabolic genes involved in lysine catabolism and fatty acid synthesis (Wu et al., 2015).

**FIGURE 4:**
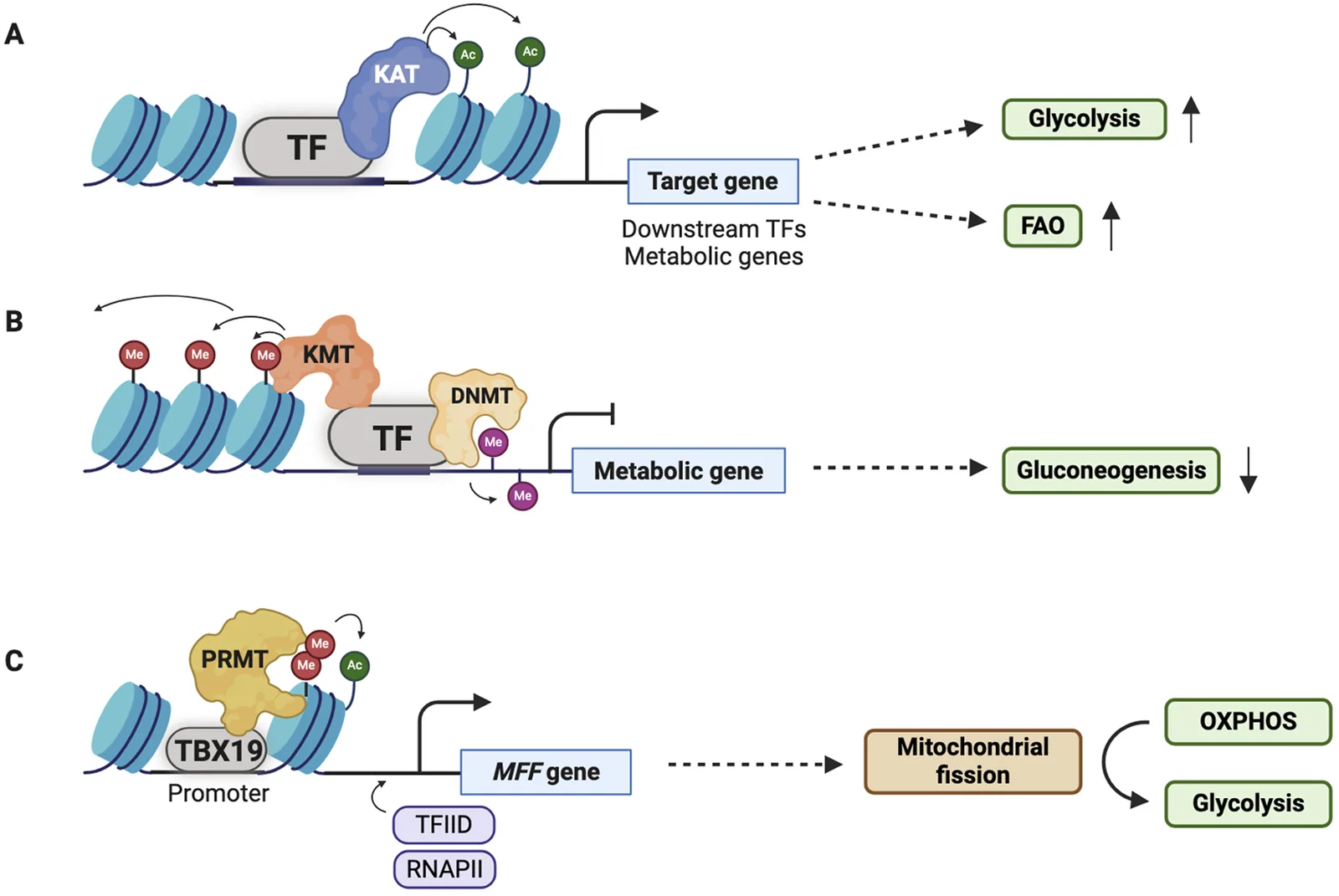
Transcription factors (TFs) can modulate metabolic adaptation of CSCs. **(A,B)** Stem cell pluripotency TFs, EMT TFs, and hypoxia-inducible factor-1 (HIF-1) activate or repress expression of metabolic genes through epigenetic modifications in CSCs. Typically, metabolic genes involved in glycolysis and fatty acid oxidation (FAO) or downstream TFs regulating metabolic adaptation are activated while metabolic genes involved in gluconeogenesis and oxidative phosphorylation (OXPHOS) are repressed. **(C)** T-box transcription factor 19 (TBX19), a TF, leads to a metabolic transition from OXPHOS to glycolysis via mitochondrial fission activated by expression of *MFF*. RNAPII; RNA polymerase II.

The tumor microenvironment influences CSC stemness, in part, through the reprogramming of tumor cell metabolism (Zheng et al., 2021; Elia and Haigis, 2021). Hypoxia, a common characteristic of the tumor microenvironment, epigenetically controls metabolic pathways primarily by activating the hypoxia-inducible factor 1 (HIF-1) (Chen et al., 2023). HIF-1 is a heterodimeric TF consisting of HIF-1α and HIF-1β. Upon activation, HIF-1α interacts with p300/CBP (CREB-binding protein), which in turn acetylates histones and recruits other TFs for gene activation (Gu et al., 2001; Lu et al., 2021). Recruited TFs such as OCT3/4, NANOG, SOX2, Snail, and TWIST contribute to the metabolic adaptation of CSCs by upregulating the expression of metabolic genes and/or their regulators (Zhang et al., 2020; Wang Y. et al., 2013; Jeter et al., 2015). Furthermore, HIF-1α can directly upregulate several glycolytic genes to meet high metabolic demands of CSCs, including GLUT1/2, hexokinases, aldolases, phosphoglycerate kinase 1, pyruvate kinase M, and enolases (Taylor and Scholz, 2022; Semenza, 2003).

Another interesting mechanism that strengthens the self-renewal capacity of CSCs is via T-box transcription factor 19 (TBX19)-mediated metabolic adaptation (Figure 4). TBX19 epigenetically regulates mitochondrial fission-mediated metabolic adaptation in liver CSCs (Tang et al., 2021). Specifically, TBX19 interacts with PRMT1, which increases the H4R3me2a/H3K9ac-mediated transcription of the mitochondrial fission factor (MFF) gene. The TBX19/MFF axis induces mitochondrial fission, which promotes the metabolic switch from OXPHOS to glycolysis. This metabolic switch, which involves mitochondrial fission, prevents ROS-mediated OCT4 degradation, thereby maintaining the stemness of liver CSCs.

### 4.2 Histone modification

Histone modifications can induce chromatin structural rearrangements and recruit chromatin factors, thereby affecting gene expression (Zentner and Henikoff, 2013). A wide array of histone modifications has been identified (Bannister and Kouzarides, 2011). Among these, histone acetylation/deacetylation and histone methylation/demethylation are the most well-characterized. We focus on how these two histone modification modules contribute to CSC metabolic adaptation.

#### 4.2.1 Histone acetylation/deacetylation

Histone acetyltransferases (HATs) and histone deacetylases (HDACs) catalyze the addition or removal, respectively, of acetyl groups from the lysine residues of target proteins (Yang and Seto, 2007). HATs are also known as lysine acetyltransferases. HATs have six families and 17 subtypes in humans, and 18 HDACs are grouped into two families and four classes (Seto and Yoshida, 2014; Wapenaar and Dekker, 2016). Classes I and II HDACs require Zn^2+^ for deacetylation. Class III HDACs belong to the silent information regulator 2 (Sir2)-related protein family (Sirtuins, SIRTs) that contains SIRT1–7. The sirtuin family HDACs use NAD^+^ as the cofactor. Class IV HDAC includes HDAC11.

Acetylation and deacetylation of the ε-amino group of the lysine residue of histones directly affect the transcription of metabolic genes (Figure 5) (Verdone et al., 2006). In HCC, HDAC11 contributes to the maintenance of cancer stemness by regulating glycolysis (Bi et al., 2021). HDAC11 deacetylates H3K9ac in the serine threonine kinase 11 (STK11) promoter region, leading to its suppression. Since STK11 activates the AMP-activated protein kinase signaling pathway, which negatively regulates glucose uptake and glycolysis, HDAC11-mediated downregulation of STK11 promotes glycolysis, thereby maintaining the stemness of HCC CSCs. SIRT6 can deacetylate H3K9ac and H3K56ac, which negatively regulates the transcription of HIF-1α-dependent glycolytic genes, including GLUT1, pyruvate dehydrogenase kinase 1, and lactate dehydrogenase A (Zhong et al., 2010). In a colorectal cancer model with an *Apc* mutation, an increase in glycolysis due to the loss of *Sirt6* can drive colorectal cancer initiation via aberrant histone acetylation (Sebastián et al., 2012). Furthermore, Sebastian et al. identified a subset of cancer cells with high glycolytic activity as quiescent tumor-initiating cells with better defense against oxidative stress (Sebastian et al., 2022). In line with these findings, in head and neck squamous cell carcinoma, CD34^+^ CSCs with *Sirt6* loss display a high glycolytic phenotype, antioxidant protection, and nucleotide synthesis that collectively confers stemness and malignancy (Choi et al., 2021).

**FIGURE 5:**
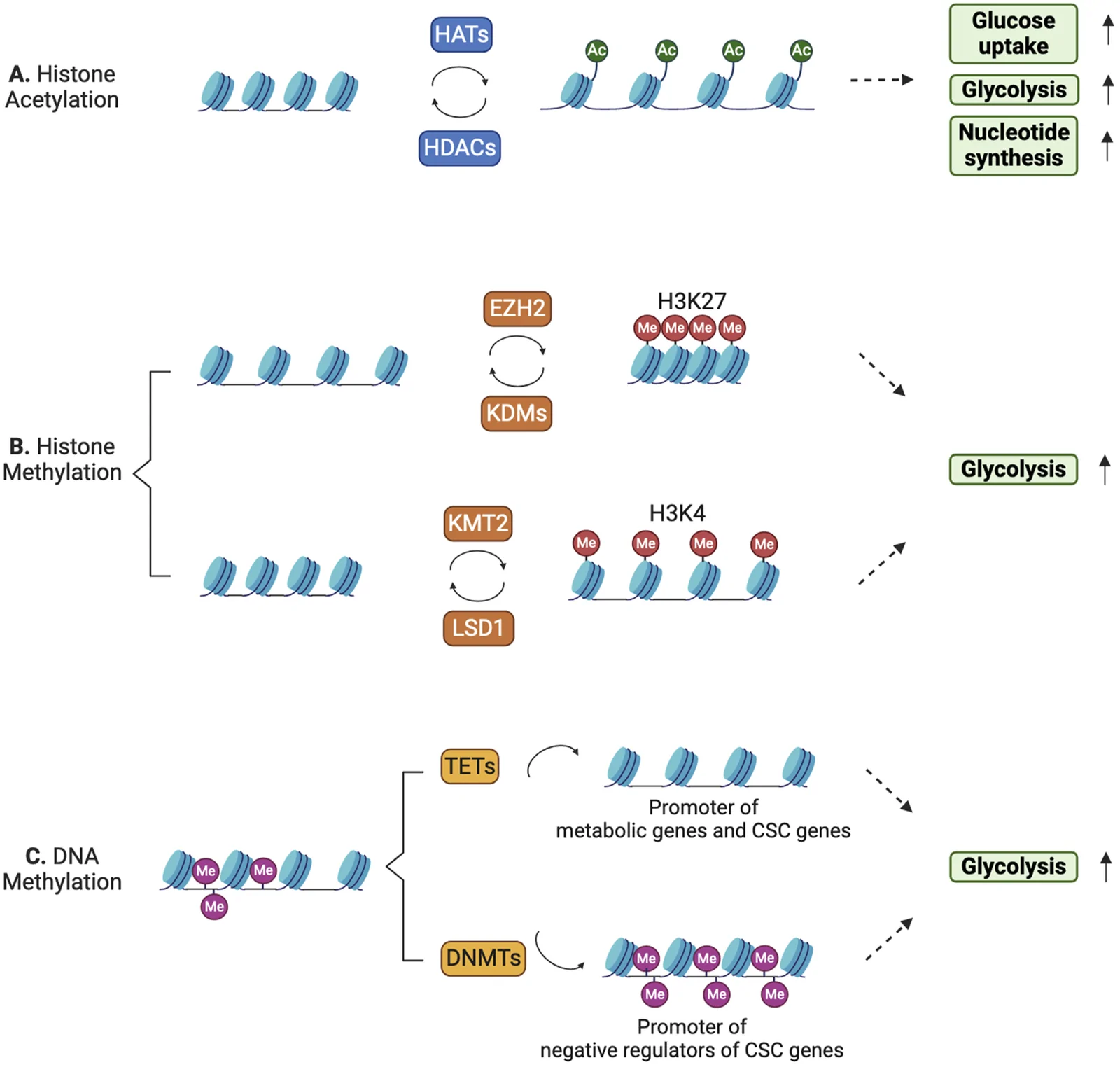
Histone and DNA modifications can dynamically control metabolic reprogramming in CSCs. **(A)** Histone acetylation/deacetylation patterns dynamically regulate gene expression in CSCs to meet their high energy demands. **(B)** Histone methylation leads to either gene repression or activation depending on the histone residues that are methylated. **(C)** DNA methylation at the promoter regions of metabolic genes and CSC-related genes is dynamically regulated.

Some HDACs have non-histone targets (Bannister and Kouzarides, 2011). CSCs leverage this noncanonical activity of HDACs for metabolic rewiring. For example, SIRT1 can deacetylate transcription coactivator PGC-1α, leading to increased expression of mitochondrial genes in chronic myeloid leukemia (CML) stem cells (Abraham et al., 2019). Upregulation of mitochondrial gene expression enhances mitochondrial activity and OXPHOS in response to the energy demands of CML stem cells. These findings underscore the multiple levels of metabolic control by epigenetic regulators.

#### 4.2.2 Histone methylation/demethylation

Histone lysine residues are methylated and demethylated by KMTs and KDMs, respectively (Dimitrova et al., 2015; Husmann and Gozani, 2019). Up to three methyl groups can be added to the ε-nitrogen of lysine residues. Canonical lysine methylation sites were found in the N-terminal tails of histones H3 and H4. Dynamic regulation of histone methylation patterns and residues is critical for the epigenetic regulation of transcription (Hyun et al., 2017).

Enhancer of zeste homolog 2 (EZH2), a catalytic subunit of polycomb repressive complex 2, mediates the trimethylation of H3K27 (H3K27me3) (Duan et al., 2020). This histone modification is associated with the repression of gene expression. Additionally, EZH2 plays an essential role in stem cell fate decisions during development (Lee et al., 2022). The aberrant upregulation of EZH2 expression has been observed in CSCs across various cancer types (Wen et al., 2017), suggesting its involvement in CSC features (Figure 5). A recent study suggested that EZH2 epigenetically promotes c-Myc-mediated glycolysis and thus tumor cell proliferation, invasion, and stemness in pancreatic cancer (Zhai et al., 2021). Specifically, EZH2 silences the expression of long non-coding RNA LINC00261, which subsequently releases IGF2BP1 from its sequestration and thus stabilizing c-Myc RNA. Upregulation of c-Myc activates target genes that regulate CSC identity.

H3K4 methylation is associated with active transcription and is catalyzed by the KMT2 protein family (Van et al., 2024). H3K4 di-methylation (H3K4me2) and H3K4 tri-methylation (H3K4me3) are enriched in leukemia stem cells (LSCs) expressing c-Kit (Wong et al., 2015). This H3K4 methylation pattern was positively correlated with the expression of metabolic genes crucial for maintaining LSC potential (Somervaille et al., 2009; Wong et al., 2015), suggesting that the H3K4 methylation pattern is associated with the transcriptional program of metabolic genes that potentially govern CSC maintenance (Figure 5).

Epigenetic regulators that affect methionine metabolism can alter the H3K4 methylation states, and thus induce the transcription of CSC-related genes. In triple-negative breast cancer, aldehyde dehydrogenase^+^ CSCs express higher levels of EMSY, a proto-oncogene, than do non-CSCs (Liu et al., 2024). Overexpression of EMSY correlates with poor prognosis, tumorigenesis, and CSC self-renewal. EMSY promotes methionine metabolism and increases SAM and S-adenosylhomocysteine levels. Elevated SAM levels fuel H3K4me2 and H3K4me3, thereby driving the upregulation of CSC-related genes such as *NANOG*, *POU5F1*, and *SOX2*. Additionally, EMSY-mediated H3K4me2 and H3K4me3 are maintained by the competitive binding of EMSY to the JmjC domain of KDM5B, demethylating H3K4 methylation (Liu et al., 2024).

### 4.3 DNA methylation

DNA methylation typically refers to the methylation of 5-carbon cytosine (5-mC) in CpG dinucleotide repeats found in the promoter regions of most mammalian genes (Moore et al., 2013). DNA methylation is catalyzed by DNMTs (Lyko, 2018), of which DNMT1, DNMT3A, and DNMT3B are canonical DNMTs responsible for 5-mC. DNA methylation is reversed by TET enzymes that convert 5-mC to 5-hydroxymethylcytosine (Lyko, 2018). The counteractive actions of DNMTs and TETs dynamically shape DNA methylation patterns. A recent study has shown that TET3 is overexpressed in LSCs in AML and promotes the expression of glycolytic genes and stem cell signature genes (Pulikkottil et al., 2022). The list includes hexokinase 1/2 and enolase 2, which are involved in glucose metabolism, and HES1 and CCND1, which are associated with early myeloid progenitor gene signatures. Overexpression of TET3 in normal human hematopoietic stem and progenitors disrupts normal hematopoietic differentiation, suggesting that TET3 maintains distinct features of LSCs in AML (Pulikkottil et al., 2022). Zinc finger DHHC-type containing 1 (ZDHHC1, ZNF377) plays a tumor-suppressive function by downregulating stemness-related TFs and providing hostile metabolic conditions for tumor growth (Le et al., 2020). Multiple cancers downregulate ZDHHC1 expression by methylating its promoter region (Le et al., 2020). The expression of stem cell pluripotency TFs and metabolic genes that maintain CSC potential are dynamically regulated at the DNA methylation level (Figure 5).

## 5 Discussion

Understanding the mechanisms underlying CSC emergence and their ability to self-renew and differentiate has clinical implications and therapeutic potential, given their association with metastasis, therapy resistance, and tumor relapse. Over the past decade, studies have demonstrated an intricate interplay between metabolic pathways and the epigenetic regulation of CSCs. Alterations in metabolic pathways enable CSCs to generate metabolites that reshape the epigenetic landscape and drive transcriptional reprogramming, which is essential for sustaining CSC features. Conversely, epigenetic regulators target metabolic genes and regulators of metabolic genes, preferentially redirecting metabolic pathways toward glycolysis and FAO from OXPHOS. In this section, we discuss the outstanding questions to be answered in this field with the current technological advances.

Recently, acyl-CoA, in addition to acetyl-CoA, has been shown to serve as a direct substrate for generating epigenetic marks that involve distinct functions in gene expression (Simithy et al., 2017). A repertoire of acyl-CoA includes propionyl, butyryl, β -hydroxybutyryl, crotonyl, succinyl, malonyl, glutaryl, and even long-chain fatty acyl groups bound to coenzyme A, capable of generating histone lysine acylation and subsequent signaling to transcriptional activation (Sabari et al., 2017). To date, no studies have investigated how acyl-CoA alters epigenetic states during CSC maintenance. Since diverse metabolites are mainly derived from lipid metabolic pathways, understanding their functional roles in regulating CSC properties through protein lysine modification mechanisms will be of considerable interest. In addition, FAD, a common dietary supplement known as vitamin B_2_ (or riboflavin), is a key cofactor of LSD1-mediated lysine demethylation. Its impact on histone (de) methylation and, in turn, CSC characteristics, remains to be explored.

The influence of metabolism on chromatin accessibility and chromatin topology remains another compelling area yet to be investigated in CSCs. The key components of the nucleosome remodeler SWI/SNF (BAF) complex are dependent on the supply of ATP to perform their enzymatic functions (Wilson and Roberts, 2011), which raises questions about how ATP availability affects the activity of the SWI/SNF complex and therefore CSC properties. The seminal paper by Bernstein and colleagues found that DNA hypermethylation in IDH mutant gliomas reduces CCCTC-binding factor (CTCF) binding and presents an associated decrease in insulator function, leading to an aberrant activation of *PDGFRA* oncogene (Flavahan et al., 2016). This paper highlights how chromosomal topology can be dysregulated in cancer under the influence of the oncometabolite. In line with it, poly (ADP-ribose) ylation (PARylation), mediated by PARP with NAD^+^ as a cofactor, is a well-known protein modification in the DNA damage response. In addition, PARP1 can regulate transcription, DNA methylation, and the function of CTCF insulator protein via PARylation (Beneke, 2012). Recently, PARylation of CTCF has been shown to affect its insulator function, thereby directly affecting the proper expression of tumor-suppressor genes such as CDH1, p16, and p19ARF (Farrar et al., 2010; Witcher and Emerson, 2009). To date, comprehensive understanding of how the functional interactions between PARP1, PAR, and CTCF can modulate the key characteristics of CSCs due to the dysregulation of NAD^+^ metabolism is lacking. Notably, in succinate dehydrogenase deficient gastrointestinal stromal tumors, global DNA hypermethylation-mediated, defective CTCF binding results in alterations in chromatin topology. The altered genome structure allows the activation of the *FGF4* oncogene by interacting with enhancers (Flavahan et al., 2019). This will be another exciting area that needs future investigation in the context of CSCs, as deficiency in key metabolic enzymes affects the chromatin topology that can drive tumorigenesis.

Furthermore, m^6^A modification of RNA can influence gene expression (Jiang et al., 2021). This RNA modification process involves m^6^A addition and removal by m^6^A methyltransferases and demethylases, respectively. Key m^6^A methyltransferases include METTL3/14/16, RBM15/15B, ZC3H3, and VIRMA, whereas demethylases include FTO and ALKBH5. METTL3 directly interacts with and modifies the transcription of metabolic genes, including HK2 and GLUT1 (Shen et al., 2020). The m^6^A modification of these transcripts is recognized by readers such as IGF2BP2 or IGFBP2/3, triggering the activation of the glycolysis pathway. This activated glycolytic pathway provides essential sustenance for tumor cell growth. As the relationship between RNA methylation/demethylation and metabolic gene regulation has recently been established, future studies to understand how their interplay drives CSC characteristics should stimulate and expand this research area.

Considering the phenotypic plasticity of CSCs, the interplay between metabolic pathways and epigenetic regulation is dynamically orchestrated, and shapes their characteristics. This interplay can be controlled by external signals received by CSCs, such as the Notch, Hedgehog, and Wnt signaling pathways (Yuan et al., 2024; Takebe et al., 2015). Therefore, investigating how external signals modulate the interaction between metabolism and epigenetic regulation in CSCs will be interesting, albeit challenging. Understanding the distinctions between these signaling pathways in CSCs and normal stem cells and their effect on metabolic enzymes and epigenetic regulators constitutes crucial research questions.

Single-cell-based techniques, such as single-cell RNA-sequencing, single-cell epigenomic profiling, and single-cell metabolomics, in addition to spatially resolved metabolite analyses with cellular resolution (e.g., matrix-assisted laser desorption/ionization-mass spectrometry imaging), offer unprecedented resolution for dissecting the heterogeneity within CSC populations (Vegliante et al., 2022). The identification of metabolically and epigenetically distinct CSC subpopulations can enable us to uncover *bona fide* CSCs associated with cancer stemness (Yuan et al., 2022; Choi et al., 2021). Additionally, single-cell techniques can provide insights into how different cell types within CSC niches affect the metabolic states and epigenetic landscapes of CSCs. Technical advancements that enable the simultaneous capture of metabolic and epigenetic states will further deepen our understanding of the interplay between metabolism and epigenetic regulation in CSC biology.

Notably, CSCs possess the unique ability to initiate and propagate malignant tumors. Their pivotal role in tumors stems from their cellular plasticity. Furthermore, CSCs are capable of self-renewal and differentiation and can dynamically change their fate in response to intrinsic and extrinsic changes, similar to their normal stem cell counterparts. Cellular plasticity is partially achieved via metabolic and epigenetic reprogramming. Markedly, both engage in a complex interplay and fine-tune cellular states. Although various methods of communication between metabolic pathways and epigenetic regulation have been identified in CSCs, further studies are needed to comprehensively understand their interplay in CSCs. Such studies could explore how metabolites, including acyl-CoA and FAD, that are yet to be examined, contribute to epigenetic alterations. Such studies can also address the effects of metabolic reprogramming on spatiotemporal genome organization and identify new pathways of epigenetic influence on metabolic regulation, such as RNA modifications. Another pivotal research direction can be to examine how CSC-associated signaling pathways govern the interplay between metabolic and epigenetic reprogramming.

Intratumor heterogeneity has led to a model in which a tumor subpopulation, now considered CSCs, is responsible for metastasis, drug resistance, and tumor relapse. However, CSCs have been identified based on their functional ability to drive tumor initiation and progression or the expression of CSC biomarkers, such as CD44, CD90, and CD133. Recent advances in single-cell-based approaches for metabolic and epigenomic profiling can enable the identification of *bona fide* CSCs, presenting a promising opportunity for complete cancer remission.

## References

[B1] Abraham A. Qiu S. Chacko B. K. Li H. Paterson A. He J. (2019). SIRT1 regulates metabolism and leukemogenic potential in CML stem cells. J. Clin. Investigation 129, 2685–2701. 10.1172/jci127080 PMC6597223 31180336

[B2] Agathocleous M. Meacham C. E. Burgess R. J. Piskounova E. Zhao Z. Crane G. M. (2017). Ascorbate regulates haematopoietic stem cell function and leukaemogenesis. Nature 549, 476–481. 10.1038/nature23876 PMC5910063 28825709

[B3] Al-Hajj M. Wicha M. S. Benito-Hernandez A. Morrison S. J. Clarke M. F. (2003). Prospective identification of tumorigenic breast cancer cells. Proc. Natl. Acad. Sci. U. S. A. 100, 3983–3988. 10.1073/pnas.0530291100 PMC153034 12629218

[B4] Altea-Manzano P. Doglioni G. Liu Y. Cuadros A. M. Nolan E. Fernández-García J. (2023). A palmitate-rich metastatic niche enables metastasis growth via p65 acetylation resulting in pro-metastatic NF-κB signaling. Nat. Cancer 4, 344–364. 10.1038/s43018-023-00513-2 PMC7615234 36732635

[B5] Baksh S. C. Todorova P. K. Gur-Cohen S. Hurwitz B. Ge Y. Novak J. S. S. (2020). Extracellular serine controls epidermal stem cell fate and tumour initiation. Nat. Cell Biol. 22, 779–790. 10.1038/s41556-020-0525-9 PMC7343604 32451440

[B6] Bannister A. J. Kouzarides T. (2011). Regulation of chromatin by histone modifications. Cell Res. 21, 381–395. 10.1038/cr.2011.22 PMC3193420 21321607

[B7] Batlle E. Clevers H. (2017). Cancer stem cells revisited. Nat. Med. 23, 1124–1134. 10.1038/nm.4409 28985214

[B8] Beneke S. (2012). Regulation of chromatin structure by poly(ADP-ribosyl)ation. Front. Genet. 3, 169. 10.3389/fgene.2012.00169 PMC3432497 22969794

[B9] Bi L. Ren Y. Feng M. Meng P. Wang Q. Chen W. (2021). HDAC11 regulates glycolysis through the LKB1/AMPK signaling pathway to maintain hepatocellular carcinoma stemness. Cancer Res. 81, 2015–2028. 10.1158/0008-5472.can-20-3044 33602787

[B10] Bonnet D. Dick J. E. (1997). Human acute myeloid leukemia is organized as a hierarchy that originates from a primitive hematopoietic cell. Nat. Med. 3, 730–737. 10.1038/nm0797-730 9212098

[B11] Boon R. Silveira G. G. Mostoslavsky R. (2020). Nuclear metabolism and the regulation of the epigenome. Nat. Metab. 2, 1190–1203. 10.1038/s42255-020-00285-4 33046909

[B12] Carey B. W. Finley L. W. Cross J. R. Allis C. D. Thompson C. B. (2015). Intracellular α-ketoglutarate maintains the pluripotency of embryonic stem cells. Nature 518, 413–416. 10.1038/nature13981 PMC4336218 25487152

[B13] Chaudhary A. Raza S. S. Haque R. (2023). Transcriptional factors targeting in cancer stem cells for tumor modulation. Seminars Cancer Biol. 88, 123–137. 10.1016/j.semcancer.2022.12.010 36603792

[B14] Chen C. L. Uthaya Kumar D. B. Punj V. Xu J. Sher L. Tahara S. M. (2016). NANOG metabolically reprograms tumor-initiating stem-like cells through tumorigenic changes in oxidative phosphorylation and fatty acid metabolism. Cell Metab. 23, 206–219. 10.1016/j.cmet.2015.12.004 PMC4715587 26724859

[B15] Chen Z. Han F. Du Y. Shi H. Zhou W. (2023). Hypoxic microenvironment in cancer: molecular mechanisms and therapeutic interventions. Signal Transduct. Target. Ther. 8, 70. 10.1038/s41392-023-01332-8 PMC9935926 36797231

[B16] Choi J. E. Mostoslavsky R. (2014). Sirtuins, metabolism, and DNA repair. Curr. Opin. Genet. and Dev. 26, 24–32. 10.1016/j.gde.2014.05.005 PMC4254145 25005742

[B17] Choi J. E. Sebastian C. Ferrer C. M. Lewis C. A. Sade-Feldman M. Lasalle T. (2021). A unique subset of glycolytic tumour-propagating cells drives squamous cell carcinoma. Nat. Metab. 3, 182–195. 10.1038/s42255-021-00350-6 PMC7954080 33619381

[B18] Chowdhury R. Yeoh K. K. Tian Y. M. Hillringhaus L. Bagg E. A. Rose N. R. (2011). The oncometabolite 2-hydroxyglutarate inhibits histone lysine demethylases. EMBO Rep. 12, 463–469. 10.1038/embor.2011.43 PMC3090014 21460794

[B19] Cimmino L. Dolgalev I. Wang Y. Yoshimi A. Martin G. H. Wang J. (2017). Restoration of TET2 function blocks aberrant self-renewal and leukemia progression. Cell 170, 1079–1095.e20. 10.1016/j.cell.2017.07.032 PMC5755977 28823558

[B20] Couto J. P. Vulin M. Jehanno C. Coissieux M. M. Hamelin B. Schmidt A. (2023). Nicotinamide N-methyltransferase sustains a core epigenetic program that promotes metastatic colonization in breast cancer. EMBO J. 42, e112559. 10.15252/embj.2022112559 PMC10308372 37259596

[B21] Dagogo-Jack I. Shaw A. T. (2018). Tumour heterogeneity and resistance to cancer therapies. Nat. Rev. Clin. Oncol. 15, 81–94. 10.1038/nrclinonc.2017.166 29115304

[B22] Dang L. White D. W. Gross S. Bennett B. D. Bittinger M. A. Driggers E. M. (2009). Cancer-associated IDH1 mutations produce 2-hydroxyglutarate. Nature 462, 739–744. 10.1038/nature08617 PMC2818760 19935646

[B23] Demicco M. Liu X. Z. Leithner K. Fendt S. M. (2024). Metabolic heterogeneity in cancer. Nat. Metab. 6, 18–38. 10.1038/s42255-023-00963-z 38267631

[B24] Dimitrova E. Turberfield A. H. Klose R. J. (2015). Histone demethylases in chromatin biology and beyond. EMBO Rep. 16, 1620–1639. 10.15252/embr.201541113 PMC4687429 26564907

[B25] Dong C. Yuan T. Wu Y. Wang Y. Fan T. W. Miriyala S. (2013). Loss of FBP1 by Snail-mediated repression provides metabolic advantages in basal-like breast cancer. Cancer Cell 23, 316–331. 10.1016/j.ccr.2013.01.022 PMC3703516 23453623

[B26] Duan R. Du W. Guo W. (2020). EZH2: a novel target for cancer treatment. J. Hematol. Oncol. 13, 104. 10.1186/s13045-020-00937-8 PMC7385862 32723346

[B27] Easwaran H. Tsai H. C. Baylin S. B. (2014). Cancer epigenetics: tumor heterogeneity, plasticity of stem-like states, and drug resistance. Mol. Cell 54, 716–727. 10.1016/j.molcel.2014.05.015 PMC4103691 24905005

[B28] Elia I. Haigis M. C. (2021). Metabolites and the tumour microenvironment: from cellular mechanisms to systemic metabolism. Nat. Metab. 3, 21–32. 10.1038/s42255-020-00317-z PMC8097259 33398194

[B29] Etchegaray J. P. Mostoslavsky R. (2016). Interplay between metabolism and epigenetics: a nuclear adaptation to environmental changes. Mol. Cell 62, 695–711. 10.1016/j.molcel.2016.05.029 PMC4893201 27259202

[B30] Farrar D. Rai S. Chernukhin I. Jagodic M. Ito Y. Yammine S. (2010). Mutational analysis of the poly(ADP-ribosyl)ation sites of the transcription factor CTCF provides an insight into the mechanism of its regulation by poly(ADP-ribosyl)ation. Mol. Cell. Biol. 30, 1199–1216. 10.1128/mcb.00827-09 PMC2820893 20038529

[B31] Faubert B. Solmonson A. Deberardinis R. J. (2020). Metabolic reprogramming and cancer progression. Science 368, eaaw5473. 10.1126/science.aaw5473 PMC7227780 32273439

[B32] Ferrie J. J. Karr J. P. Tjian R. Darzacq X. (2022). “Structure”-function relationships in eukaryotic transcription factors: the role of intrinsically disordered regions in gene regulation. Mol. Cell 82, 3970–3984. 10.1016/j.molcel.2022.09.021 36265487

[B33] Figueroa M. E. Abdel-Wahab O. Lu C. Ward P. S. Patel J. Shih A. (2010). Leukemic IDH1 and IDH2 mutations result in a hypermethylation phenotype, disrupt TET2 function, and impair hematopoietic differentiation. Cancer Cell 18, 553–567. 10.1016/j.ccr.2010.11.015 PMC4105845 21130701

[B34] Flavahan W. A. Drier Y. Johnstone S. E. Hemming M. L. Tarjan D. R. Hegazi E. (2019). Altered chromosomal topology drives oncogenic programs in SDH-deficient GISTs. Nature 575, 229–233. 10.1038/s41586-019-1668-3 PMC6913936 31666694

[B35] Flavahan W. A. Drier Y. Liau B. B. Gillespie S. M. Venteicher A. S. Stemmer-Rachamimov A. O. (2016). Insulator dysfunction and oncogene activation in IDH mutant gliomas. Nature 529, 110–114. 10.1038/nature16490 PMC4831574 26700815

[B36] Ginestier C. Hur M. H. Charafe-Jauffret E. Monville F. Dutcher J. Brown M. (2007). ALDH1 is a marker of normal and malignant human mammary stem cells and a predictor of poor clinical outcome. Cell Stem Cell 1, 555–567. 10.1016/j.stem.2007.08.014 PMC2423808 18371393

[B37] Gu J. Milligan J. Huang L. E. (2001). Molecular mechanism of hypoxia-inducible factor 1α-p300 interaction. J. Biol. Chem. 276, 3550–3554. 10.1074/jbc.m009522200 11063749

[B38] Gujar A. D. Le S. Mao D. D. Dadey D. Y. Turski A. Sasaki Y. (2016). An NAD+-dependent transcriptional program governs self-renewal and radiation resistance in glioblastoma. Proc. Natl. Acad. Sci. U. S. A. 113, E8247–E8256. 10.1073/pnas.1610921114 PMC5187672 27930300

[B39] Hanahan D. (2022). Hallmarks of cancer: new dimensions. Cancer Discov. 12, 31–46. 10.1158/2159-8290.cd-21-1059 35022204

[B40] Husmann D. Gozani O. (2019). Histone lysine methyltransferases in biology and disease. Nat. Struct. Mol. Biol. 26, 880–889. 10.1038/s41594-019-0298-7 PMC6951022 31582846

[B41] Hyun K. Jeon J. Park K. Kim J. (2017). Writing, erasing and reading histone lysine methylations. Exp. Mol. Med. 49, e324. 10.1038/emm.2017.11 PMC6130214 28450737

[B42] Izzo L. T. Affronti H. C. Wellen K. E. (2021). The bidirectional relationship between cancer epigenetics and metabolism. Annu. Rev. Cancer Biol. 5, 235–257. 10.1146/annurev-cancerbio-070820-035832 PMC8186467 34109280

[B43] Jeter C. R. Yang T. Wang J. Chao H. P. Tang D. G. (2015). Concise review: NANOG in cancer stem cells and tumor development: an update and outstanding questions. Stem Cells 33, 2381–2390. 10.1002/stem.2007 PMC4509798 25821200

[B44] Jiang X. Liu B. Nie Z. Duan L. Xiong Q. Jin Z. (2021). The role of m6A modification in the biological functions and diseases. Signal Transduct. Target. Ther. 6, 74. 10.1038/s41392-020-00450-x PMC7897327 33611339

[B45] Jung J. Kim L. J. Wang X. Wu Q. Sanvoranart T. Hubert C. G. (2017). Nicotinamide metabolism regulates glioblastoma stem cell maintenance. JCI Insight 2, e90019. 10.1172/jci.insight.90019 PMC5436539 28515364

[B46] Killian J. K. Kim S. Y. Miettinen M. Smith C. Merino M. Tsokos M. (2013). Succinate dehydrogenase mutation underlies global epigenomic divergence in gastrointestinal stromal tumor. Cancer Discov. 3, 648–657. 10.1158/2159-8290.cd-13-0092 PMC4135374 23550148

[B47] Kim J. Deberardinis R. J. (2019). Mechanisms and implications of metabolic heterogeneity in cancer. Cell Metab. 30, 434–446. 10.1016/j.cmet.2019.08.013 PMC6730674 31484055

[B48] Klose R. J. Kallin E. M. Zhang Y. (2006). JmjC-domain-containing proteins and histone demethylation. Nat. Rev. Genet. 7, 715–727. 10.1038/nrg1945 16983801

[B49] Kooistra S. M. Helin K. (2012). Molecular mechanisms and potential functions of histone demethylases. Nat. Rev. Mol. Cell Biol. 13, 297–311. 10.1038/nrm3327 22473470

[B50] Kreso A. Dick J. E. (2014). Evolution of the cancer stem cell model. Cell Stem Cell 14, 275–291. 10.1016/j.stem.2014.02.006 24607403

[B51] Lapidot T. Sirard C. Vormoor J. Murdoch B. Hoang T. Caceres-Cortes J. (1994). A cell initiating human acute myeloid leukaemia after transplantation into SCID mice. Nature 367, 645–648. 10.1038/367645a0 7509044

[B52] Le X. Mu J. Peng W. Tang J. Xiang Q. Tian S. (2020). DNA methylation downregulated ZDHHC1 suppresses tumor growth by altering cellular metabolism and inducing oxidative/ER stress-mediated apoptosis and pyroptosis. Theranostics 10, 9495–9511. 10.7150/thno.45631 PMC7449911 32863941

[B53] Lee S. H. Li Y. Kim H. Eum S. Park K. Lee C. H. (2022). The role of EZH1 and EZH2 in development and cancer. BMB Rep. 55, 595–601. 10.5483/BMBRep.2022.55.12.174 PMC9813427 36476271

[B54] Letouzé E. Martinelli C. Loriot C. Burnichon N. Abermil N. Ottolenghi C. (2013). SDH mutations establish a hypermethylator phenotype in paraganglioma. Cancer Cell 23, 739–752. 10.1016/j.ccr.2013.04.018 23707781

[B55] Lisowski P. Kannan P. Mlody B. Prigione A. (2018). Mitochondria and the dynamic control of stem cell homeostasis. EMBO Rep. 19, e45432. 10.15252/embr.201745432 PMC5934764 29661859

[B56] Liu C. C. Chen L. Cai Y. W. Chen Y. F. Liu Y. M. Zhou Y. J. (2024). Targeting EMSY-mediated methionine metabolism is a potential therapeutic strategy for triple-negative breast cancer. Cell Rep. Med. 5, 101396. 10.1016/j.xcrm.2024.101396 PMC10897545 38290515

[B57] Loo S. Y. Toh L. P. Xie W. H. Pathak E. Tan W. Ma S. (2021). Fatty acid oxidation is a druggable gateway regulating cellular plasticity for driving metastasis in breast cancer. Sci. Adv. 7, eabh2443. 10.1126/sciadv.abh2443 PMC8494440 34613780

[B58] Losman J. A. Koivunen P. Kaelin W. G. (2020). 2-Oxoglutarate-dependent dioxygenases in cancer. Nat. Rev. Cancer 20, 710–726. 10.1038/s41568-020-00303-3 33087883

[B59] Losman J. A. Looper R. E. Koivunen P. Lee S. Schneider R. K. Mcmahon C. (2013). (R)-2-hydroxyglutarate is sufficient to promote leukemogenesis and its effects are reversible. Science 339, 1621–1625. 10.1126/science.1231677 PMC3836459 23393090

[B60] Lu C. Ward P. S. Kapoor G. S. Rohle D. Turcan S. Abdel-Wahab O. (2012). IDH mutation impairs histone demethylation and results in a block to cell differentiation. Nature 483, 474–478. 10.1038/nature10860 PMC3478770 22343901

[B61] Lu H. Lyu Y. Tran L. Lan J. Xie Y. Yang Y. (2021). HIF-1 recruits NANOG as a coactivator for TERT gene transcription in hypoxic breast cancer stem cells. Cell Rep. 36, 109757. 10.1016/j.celrep.2021.109757 34592152

[B62] Lucena-Cacace A. Otero-Albiol D. Jiménez-García M. P. Muñoz-Galvan S. Carnero A. (2018). *NAMPT* is a potent oncogene in colon cancer progression that modulates cancer stem cell properties and resistance to therapy through Sirt1 and PARP. Clin. Cancer Res. 24, 1202–1215. 10.1158/1078-0432.ccr-17-2575 29203587

[B63] Lyko F. (2018). The DNA methyltransferase family: a versatile toolkit for epigenetic regulation. Nat. Rev. Genet. 19, 81–92. 10.1038/nrg.2017.80 29033456

[B64] Maston G. A. Evans S. K. Green M. R. (2006). Transcriptional regulatory elements in the human genome. Annu. Rev. Genomics Hum. Genet. 7, 29–59. 10.1146/annurev.genom.7.080505.115623 16719718

[B65] Meacham C. E. Morrison S. J. (2013). Tumour heterogeneity and cancer cell plasticity. Nature 501, 328–337. 10.1038/nature12624 PMC4521623 24048065

[B66] Mentch S. J. Mehrmohamadi M. Huang L. Liu X. Gupta D. Mattocks D. (2015). Histone methylation dynamics and gene regulation occur through the sensing of one-carbon metabolism. Cell Metab. 22, 861–873. 10.1016/j.cmet.2015.08.024 PMC4635069 26411344

[B67] Miranda-Goncalves V. Lameirinhas A. Henrique R. Jeronimo C. (2018). Metabolism and epigenetic interplay in cancer: regulation and putative therapeutic targets. Front. Genet. 9, 427. 10.3389/fgene.2018.00427 PMC6190739 30356832

[B68] Moore L. D. Le T. Fan G. (2013). DNA methylation and its basic function. Neuropsychopharmacology 38, 23–38. 10.1038/npp.2012.112 PMC3521964 22781841

[B69] Moussaieff A. Rouleau M. Kitsberg D. Cohen M. Levy G. Barasch D. (2015). Glycolysis-mediated changes in acetyl-CoA and histone acetylation control the early differentiation of embryonic stem cells. Cell Metab. 21, 392–402. 10.1016/j.cmet.2015.02.002 25738455

[B70] Nassar D. Blanpain C. (2016). Cancer stem cells: basic concepts and therapeutic implications. Annu. Rev. Pathology Mech. Dis. 11, 47–76. 10.1146/annurev-pathol-012615-044438 27193450

[B71] O'brien C. A. Pollett A. Gallinger S. Dick J. E. (2007). A human colon cancer cell capable of initiating tumour growth in immunodeficient mice. Nature 445, 106–110. 10.1038/nature05372 17122772

[B72] Oskarsson T. Batlle E. Massagué J. (2014). Metastatic stem cells: sources, niches, and vital pathways. Cell Stem Cell 14, 306–321. 10.1016/j.stem.2014.02.002 PMC3998185 24607405

[B73] Pan M. Reid M. A. Lowman X. H. Kulkarni R. P. Tran T. Q. Liu X. (2016). Regional glutamine deficiency in tumours promotes dedifferentiation through inhibition of histone demethylation. Nat. Cell Biol. 18, 1090–1101. 10.1038/ncb3410 PMC5536113 27617932

[B74] Pascual G. Dominguez D. Elosua-Bayes M. Beckedorff F. Laudanna C. Bigas C. (2021). Dietary palmitic acid promotes a prometastatic memory via Schwann cells. Nature 599, 485–490. 10.1038/s41586-021-04075-0 34759321

[B75] Pavlova N. N. Thompson C. B. (2016). The emerging hallmarks of cancer metabolism. Cell Metab. 23, 27–47. 10.1016/j.cmet.2015.12.006 PMC4715268 26771115

[B76] Pérez-González A. Bévant K. Blanpain C. (2023). Cancer cell plasticity during tumor progression, metastasis and response to therapy. Nat. Cancer 4, 1063–1082. 10.1038/s43018-023-00595-y PMC7615147 37537300

[B77] Preissl S. Gaulton K. J. Ren B. (2023). Characterizing cis-regulatory elements using single-cell epigenomics. Nat. Rev. Genet. 24, 21–43. 10.1038/s41576-022-00509-1 PMC9771884 35840754

[B78] Pulikkottil A. J. Bamezai S. Ammer T. Mohr F. Feder K. Vegi N. M. (2022). TET3 promotes AML growth and epigenetically regulates glucose metabolism and leukemic stem cell associated pathways. Leukemia 36, 416–425. 10.1038/s41375-021-01390-3 34462525

[B79] Raffel S. Falcone M. Kneisel N. Hansson J. Wang W. Lutz C. (2017). BCAT1 restricts αKG levels in AML stem cells leading to IDHmut-like DNA hypermethylation. Nature 551, 384–388. 10.1038/nature24294 29144447

[B80] Ricci-Vitiani L. Lombardi D. G. Pilozzi E. Biffoni M. Todaro M. Peschle C. (2007). Identification and expansion of human colon-cancer-initiating cells. Nature 445, 111–115. 10.1038/nature05384 17122771

[B81] Rivera C. M. Ren B. (2013). Mapping human epigenomes. Cell 155, 39–55. 10.1016/j.cell.2013.09.011 PMC3838898 24074860

[B82] Rohle D. Popovici-Muller J. Palaskas N. Turcan S. Grommes C. Campos C. (2013). An inhibitor of mutant IDH1 delays growth and promotes differentiation of glioma cells. Science 340, 626–630. 10.1126/science.1236062 PMC3985613 23558169

[B83] Ryall J. G. Cliff T. Dalton S. Sartorelli V. (2015). Metabolic reprogramming of stem cell epigenetics. Cell Stem Cell 17, 651–662. 10.1016/j.stem.2015.11.012 PMC4672395 26637942

[B84] Sabari B. R. Zhang D. Allis C. D. Zhao Y. (2017). Metabolic regulation of gene expression through histone acylations. Nat. Rev. Mol. Cell Biol. 18, 90–101. 10.1038/nrm.2016.140 PMC5320945 27924077

[B85] Saggese P. Pandey A. Alcaraz M. Fung E. Hall A. Yanagawa J. (2024). Glucose deprivation promotes pseudohypoxia and dedifferentiation in lung adenocarcinoma. Cancer Res. 84, 305–327. 10.1158/0008-5472.can-23-1148 PMC10790128 37934116

[B86] Sebastian C. Ferrer C. Serra M. Choi J. E. Ducano N. Mira A. (2022). A non-dividing cell population with high pyruvate dehydrogenase kinase activity regulates metabolic heterogeneity and tumorigenesis in the intestine. Nat. Commun. 13, 1503. 10.1038/s41467-022-29085-y PMC8938512 35314684

[B87] Sebastián C. Zwaans B. M. Silberman D. M. Gymrek M. Goren A. Zhong L. (2012). The histone deacetylase SIRT6 is a tumor suppressor that controls cancer metabolism. Cell 151, 1185–1199. 10.1016/j.cell.2012.10.047 PMC3526953 23217706

[B88] Semenza G. L. (2003). Targeting HIF-1 for cancer therapy. Nat. Rev. Cancer 3, 721–732. 10.1038/nrc1187 13130303

[B89] Seto E. Yoshida M. (2014). Erasers of histone acetylation: the histone deacetylase enzymes. Cold Spring Harb. Perspect. Biol. 6, a018713. 10.1101/cshperspect.a018713 PMC3970420 24691964

[B90] Shen C. Xuan B. Yan T. Ma Y. Xu P. Tian X. (2020). m(6)A-dependent glycolysis enhances colorectal cancer progression. Mol. Cancer 19, 72. 10.1186/s12943-020-01190-w PMC7118901 32245489

[B91] Shiraki N. Shiraki Y. Tsuyama T. Obata F. Miura M. Nagae G. (2014). Methionine metabolism regulates maintenance and differentiation of human pluripotent stem cells. Cell Metab. 19, 780–794. 10.1016/j.cmet.2014.03.017 24746804

[B92] Shyh-Chang N. Locasale J. W. Lyssiotis C. A. Zheng Y. Teo R. Y. Ratanasirintrawoot S. (2013). Influence of threonine metabolism on S-adenosylmethionine and histone methylation. Science 339, 222–226. 10.1126/science.1226603 PMC3652341 23118012

[B93] Simithy J. Sidoli S. Yuan Z. F. Coradin M. Bhanu N. V. Marchione D. M. (2017). Characterization of histone acylations links chromatin modifications with metabolism. Nat. Commun. 8, 1141. 10.1038/s41467-017-01384-9 PMC5656686 29070843

[B94] Singh R. P. Jeyaraju D. V. Voisin V. Hurren R. Xu C. Hawley J. R. (2020). Disrupting mitochondrial copper distribution inhibits leukemic stem cell self-renewal. Cell Stem Cell 26, 926–937.e10. 10.1016/j.stem.2020.04.010 32416059

[B95] Singh S. K. Hawkins C. Clarke I. D. Squire J. A. Bayani J. Hide T. (2004). Identification of human brain tumour initiating cells. Nature 432, 396–401. 10.1038/nature03128 15549107

[B96] Sivanand S. Viney I. Wellen K. E. (2018). Spatiotemporal control of acetyl-CoA metabolism in chromatin regulation. Trends Biochem. Sci. 43, 61–74. 10.1016/j.tibs.2017.11.004 PMC5741483 29174173

[B97] Somervaille T. C. Matheny C. J. Spencer G. J. Iwasaki M. Rinn J. L. Witten D. M. (2009). Hierarchical maintenance of MLL myeloid leukemia stem cells employs a transcriptional program shared with embryonic rather than adult stem cells. Cell Stem Cell 4, 129–140. 10.1016/j.stem.2008.11.015 PMC2670853 19200802

[B98] Sperber H. Mathieu J. Wang Y. Ferreccio A. Hesson J. Xu Z. (2015). The metabolome regulates the epigenetic landscape during naive-to-primed human embryonic stem cell transition. Nat. Cell Biol. 17, 1523–1535. 10.1038/ncb3264 PMC4662931 26571212

[B99] Stallcup M. R. (2001). Role of protein methylation in chromatin remodeling and transcriptional regulation. Oncogene 20, 3014–3020. 10.1038/sj.onc.1204325 11420716

[B100] Strekalova E. Malin D. Weisenhorn E. M. M. Russell J. D. Hoelper D. Jain A. (2019). S-adenosylmethionine biosynthesis is a targetable metabolic vulnerability of cancer stem cells. Breast Cancer Res. Treat. 175, 39–50. 10.1007/s10549-019-05146-7 PMC6494685 30712196

[B101] Su R. Dong L. Li C. Nachtergaele S. Wunderlich M. Qing Y. (2018). R-2HG exhibits anti-tumor activity by targeting FTO/m6A/MYC/CEBPA signaling. Cell 172, 90–105.e23. 10.1016/j.cell.2017.11.031 PMC5766423 29249359

[B102] Takebe N. Miele L. Harris P. J. Jeong W. Bando H. Kahn M. (2015). Targeting Notch, Hedgehog, and Wnt pathways in cancer stem cells: clinical update. Nat. Rev. Clin. Oncol. 12, 445–464. 10.1038/nrclinonc.2015.61 PMC4520755 25850553

[B103] Tang M. Yang M. Wu G. Mo S. Wu X. Zhang S. (2021). Epigenetic induction of mitochondrial fission is required for maintenance of liver cancer-initiating cells. Cancer Res. 81, 3835–3848. 10.1158/0008-5472.can-21-0436 34049973

[B104] Taylor C. T. Scholz C. C. (2022). The effect of HIF on metabolism and immunity. Nat. Rev. Nephrol. 18, 573–587. 10.1038/s41581-022-00587-8 PMC9208707 35726016

[B105] Van H. T. Xie G. Dong P. Liu Z. Ge K. (2024). KMT2 family of H3K4 methyltransferases: enzymatic activity-dependent and -independent functions. J. Mol. Biol. 436, 168453. 10.1016/j.jmb.2024.168453 PMC10957308 38266981

[B106] Vegliante R. Pastushenko I. Blanpain C. (2022). Deciphering functional tumor states at single-cell resolution. EMBO J. 41, e109221. 10.15252/embj.2021109221 PMC8762559 34918370

[B107] Verdone L. Agricola E. Caserta M. Di Mauro E. (2006). Histone acetylation in gene regulation. Brief. Funct. Genomic Proteomic 5, 209–221. 10.1093/bfgp/ell028 16877467

[B108] Wainwright E. N. Scaffidi P. (2017). Epigenetics and cancer stem cells: unleashing, hijacking, and restricting cellular plasticity. Trends Cancer 3, 372–386. 10.1016/j.trecan.2017.04.004 PMC5506260 28718414

[B109] Wang F. Travins J. Delabarre B. Penard-Lacronique V. Schalm S. Hansen E. (2013a). Targeted inhibition of mutant IDH2 in leukemia cells induces cellular differentiation. Science 340, 622–626. 10.1126/science.1234769 23558173

[B110] Wang L. Tang Y. (2023). N6-methyladenosine (m6A) in cancer stem cell: from molecular mechanisms to therapeutic implications. Biomed. and Pharmacother. 163, 114846. 10.1016/j.biopha.2023.114846 37167725

[B111] Wang Y. Shi J. Chai K. Ying X. Zhou B. P. (2013b). The role of Snail in EMT and tumorigenesis. Curr. Cancer Drug Targets 13, 963–972. 10.2174/15680096113136660102 PMC4004763 24168186

[B112] Wang Z. Yip L. Y. Lee J. H. J. Wu Z. Chew H. Y. Chong P. K. W. (2019). Methionine is a metabolic dependency of tumor-initiating cells. Nat. Med. 25, 825–837. 10.1038/s41591-019-0423-5 31061538

[B113] Wapenaar H. Dekker F. J. (2016). Histone acetyltransferases: challenges in targeting bi-substrate enzymes. Clin. Epigenetics 8, 59. 10.1186/s13148-016-0225-2 PMC4881052 27231488

[B114] Wellen K. E. Hatzivassiliou G. Sachdeva U. M. Bui T. V. Cross J. R. Thompson C. B. (2009). ATP-citrate lyase links cellular metabolism to histone acetylation. Science 324, 1076–1080. 10.1126/science.1164097 PMC2746744 19461003

[B115] Wen Y. Cai J. Hou Y. Huang Z. Wang Z. (2017). Role of EZH2 in cancer stem cells: from biological insight to a therapeutic target. Oncotarget 8, 37974–37990. 10.18632/oncotarget.16467 PMC5514966 28415635

[B116] Wilson B. G. Roberts C. W. (2011). SWI/SNF nucleosome remodellers and cancer. Nat. Rev. Cancer 11, 481–492. 10.1038/nrc3068 21654818

[B117] Witcher M. Emerson B. M. (2009). Epigenetic silencing of the p16 tumor suppressor is associated with loss of CTCF binding and a chromatin boundary. Mol. Cell 34, 271–284. 10.1016/j.molcel.2009.04.001 PMC2723750 19450526

[B118] Wong S. H. Goode D. L. Iwasaki M. Wei M. C. Kuo H. P. Zhu L. (2015). The H3K4-methyl epigenome regulates leukemia stem cell oncogenic potential. Cancer Cell 28, 198–209. 10.1016/j.ccell.2015.06.003 PMC4536132 26190263

[B119] Wu Z. Wei D. Gao W. Xu Y. Hu Z. Ma Z. (2015). TPO-induced metabolic reprogramming drives liver metastasis of colorectal cancer CD110+ tumor-initiating cells. Cell Stem Cell 17, 47–59. 10.1016/j.stem.2015.05.016 26140605

[B120] Xiao M. Yang H. Xu W. Ma S. Lin H. Zhu H. (2012). Inhibition of α-KG-dependent histone and DNA demethylases by fumarate and succinate that are accumulated in mutations of FH and SDH tumor suppressors. Genes Dev. 26, 1326–1338. 10.1101/gad.191056.112 PMC3387660 22677546

[B121] Xu W. Yang H. Liu Y. Yang Y. Wang P. Kim S. H. (2011). Oncometabolite 2-hydroxyglutarate is a competitive inhibitor of α-ketoglutarate-dependent dioxygenases. Cancer Cell 19, 17–30. 10.1016/j.ccr.2010.12.014 PMC3229304 21251613

[B122] Yang D. Peng M. Hou Y. Qin Y. Wan X. Zhu P. (2020a). Oxidized ATM promotes breast cancer stem cell enrichment through energy metabolism reprogram-mediated acetyl-CoA accumulation. Cell Death Dis. 11, 508. 10.1038/s41419-020-2714-7 PMC7343870 32641713

[B123] Yang L. Shi P. Zhao G. Xu J. Peng W. Zhang J. (2020b). Targeting cancer stem cell pathways for cancer therapy. Signal Transduct. Target. Ther. 5, 8. 10.1038/s41392-020-0110-5 PMC7005297 32296030

[B124] Yang X. J. Seto E. (2007). HATs and HDACs: from structure, function and regulation to novel strategies for therapy and prevention. Oncogene 26, 5310–5318. 10.1038/sj.onc.1210599 17694074

[B125] Yuan S. Almagro J. Fuchs E. (2024). Beyond genetics: driving cancer with the tumour microenvironment behind the wheel. Nat. Rev. Cancer 24, 274–286. 10.1038/s41568-023-00660-9 PMC11077468 38347101

[B126] Yuan S. Stewart K. S. Yang Y. Abdusselamoglu M. D. Parigi S. M. Feinberg T. Y. (2022). Ras drives malignancy through stem cell crosstalk with the microenvironment. Nature 612, 555–563. 10.1038/s41586-022-05475-6 PMC9750880 36450983

[B127] Zentner G. E. Henikoff S. (2013). Regulation of nucleosome dynamics by histone modifications. Nat. Struct. Mol. Biol. 20, 259–266. 10.1038/nsmb.2470 23463310

[B128] Zhai S. Xu Z. Xie J. Zhang J. Wang X. Peng C. (2021). Epigenetic silencing of LncRNA LINC00261 promotes c-myc-mediated aerobic glycolysis by regulating miR-222-3p/HIPK2/ERK axis and sequestering IGF2BP1. Oncogene 40, 277–291. 10.1038/s41388-020-01525-3 PMC7808938 33122827

[B129] Zhang C. Du Z. Gao Y. Lim K. S. Zhou W. Huang H. (2024). Methionine secreted by tumor-associated pericytes supports cancer stem cells in clear cell renal carcinoma. Cell Metab. 36, 778–792.e10. 10.1016/j.cmet.2024.01.018 38378000

[B130] Zhang C. Samanta D. Lu H. Bullen J. W. Zhang H. Chen I. (2016). Hypoxia induces the breast cancer stem cell phenotype by HIF-dependent and ALKBH5-mediated m ^6^ A-demethylation of NANOG mRNA. Proc. Natl. Acad. Sci. U. S. A. 113, E2047–E2056. 10.1073/pnas.1602883113 PMC4833258 27001847

[B131] Zhang Q. Han Z. Zhu Y. Chen J. Li W. (2020). Role of hypoxia inducible factor-1 in cancer stem cells (Review). Mol. Med. Rep. 23, 1. 10.3892/mmr.2020.11655 PMC7673349 33179080

[B132] Zheng X. Yu C. Xu M. (2021). Linking tumor microenvironment to plasticity of cancer stem cells: mechanisms and application in cancer therapy. Front. Oncol. 11, 678333. 10.3389/fonc.2021.678333 PMC8273276 34262865

[B133] Zhong L. D'urso A. Toiber D. Sebastian C. Henry R. E. Vadysirisack D. D. (2010). The histone deacetylase Sirt6 regulates glucose homeostasis via Hif1alpha. Cell 140, 280–293. 10.1016/j.cell.2009.12.041 PMC2821045 20141841

